# Prognostic Impact of Aspartate Aminotransferase-to-Platelet Ratio Index and Prognostic Nutrition Index in Hepatocellular Carcinoma Patients Undergoing Resection

**DOI:** 10.3390/jcm14165665

**Published:** 2025-08-11

**Authors:** Matteo Risaliti, Valerio De Peppo, Ilenia Bartolini, Luca Tirloni, Andrea Scarinci, Irene Terrenato, Gian Luca Grazi

**Affiliations:** 1 Hepato-Pancreato-Biliary Surgery, Azienda Ospedaliero-Universitaria Careggi, Department of Clinical and Experimental Medicine, University of Florence, 50134 Florence, Italy; risalitim@aou-careggi.toscana.it (M.R.); ilenia.bartolini@gmail.com (I.B.); gianluca.grazi@unifi.it (G.L.G.); 2 Hepato-Pancreato-Biliary Surgery, IRCCS, Regina Elena National Cancer Institute, 00128 Rome, Italy; valeriodepeppo@gmail.com (V.D.P.); andrea.scarinci@ifo.it (A.S.); 3 Biostatistics and Bioinformatics Unit, IRCCS, Regina Elena National Cancer Institute, 00128 Rome, Italy; irene.terrenato@ifo.it

**Keywords:** systemic inflammatory indices, PNI, APRI score, primary malignant liver tumor, hepatocellular carcinoma, hepatic resection

## Abstract

**Background/Objectives:** Tumor-associated inflammation plays a crucial role in supporting tumorigenesis and the progression of oncological diseases. This study aimed to evaluate whether systemic inflammatory indices are associated with overall survival (OS) in patients with hepatocellular carcinoma (HCC) undergoing surgery. **Methods**: A retrospective cohort study was conducted on consecutive patients with HCC who underwent hepatic resection. Data were prospectively collected and retrospectively reviewed. The 5-year OS rate was used as the primary endpoint to stratify the values of inflammatory indices, including the neutrophil-to-lymphocyte ratio (NLR), platelet-to-lymphocyte ratio (PLR), lymphocyte-to-monocyte ratio (LMR), aspartate aminotransferase-to-neutrophil ratio index (ANRI), fibrinogen-to-albumin ratio (Fib-Alb), the systemic immune-inflammation index (SII), prognostic nutritional index (PNI), and aspartate aminotransferase-to-platelet ratio index (APRI), through receiver operating characteristic (ROC) curve analysis. The optimal albumin–bilirubin (ALBI) and platelet–ALBI (PALBI) cut-off points from the literature were also applied. **Results**: Patients included in the study were 153. The 1-, 3-, and 5-year OS rates were 81.7%, 65.2%, and 40.7%, respectively. Univariate Cox proportional hazards analysis showed that, in addition to several patient- and tumor-related characteristics and postoperative complications, elevated values of PLR, ANRI, Fib-Alb, SII, APRI, ALBI, and PALBI, as well as low PNI, were significantly associated with poorer overall survival (OS). Among these, only APRI and PNI emerged as independent prognostic factors in the multivariate analysis. **Conclusions**: PNI and APRI could serve as valuable inflammatory indices for predicting OS, helping to identify HCC patients who might benefit from hepatic resection. However, further prospective studies with larger cohorts are needed to validate the prognostic role of PNI and APRI.

## 1. Introduction

Hepatocellular carcinoma (HCC) is a highly malignant tumor with significant morbidity and mortality. It is the sixth most common cancer and the fourth leading cause of cancer-related deaths worldwide [1,2].

In most cases of HCC, there is an underlying liver disease [3]. HCC treatment options depend on various factors. The most effective curative treatment for HCC is liver transplantation (LT), though this is limited by the shortage of available organs. Hepatic resection (HR) remains the primary treatment for achieving oncological radicality with survival outcomes comparable to those of LT despite cirrhosis if patients are carefully selected [4,5]. However, only 10–37% of HCC patients are candidates for curative resection, and outcomes, such as overall survival (OS), recurrence-free survival (RFS), and disease-free survival, remain suboptimal [6]. Several factors influence prognosis, including tumor size, tumor differentiation, vascular invasion (both micro- and macro-), liver function, and surgical margin status. While liver function can be assessed preoperatively, many of these factors can be evaluated only postoperatively, limiting their use as prognostic indicators before surgery [7].

Circulating inflammatory biomarkers has the potential to reflect underlying systemic inflammation and may offer insights into the prognosis of HCC [8]. Various inflammatory indices can be easily obtained from simple, low-cost peripheral blood tests (e.g., neutrophils, lymphocytes, monocytes, and platelets) and include the neutrophil-to-lymphocyte ratio (NLR), platelet-to-lymphocyte ratio (PLR), prognostic nutritional index (PNI), lymphocyte-to-monocyte ratio (LMR), aspartate aminotransferase-to-neutrophil ratio index (ANRI), aspartate aminotransferase-to-platelet ratio index (APRI), fibrinogen-to-albumin ratio (Fib/Alb), and the systemic immune-inflammation index (SII) [9,10]. Tumor-associated inflammation is a key feature of HCC indeed [11]. In many cases, this inflammation leads to fibrosis and, over time, to cirrhosis supporting tumorigenesis and progression [11,12]. For these reasons, prognostic biomarkers could be important to predict the prognosis of HCC patients before HR.

In recent years, inflammatory markers have been studied for their prognostic value in cancers such as non-small cell lung cancer, gastric cancer, ovarian cancer, pancreatic cancer, and nasopharyngeal cancer [13,14,15,16,17]. However, there is a lack of studies evaluating which combination of these markers offers a superior prognostic value compared to individual markers alone.

Based on this background, this study aimed to comprehensively evaluate the prognostic efficacy of various systemic inflammatory indices in predicting survival outcomes following hepatic resection for hepatocellular carcinoma (HCC). The primary goal was to assess the effectiveness and prognostic significance of ten such indices, while the secondary objective was to identify the most robust and clinically useful index among them for predicting survival in this specific patient cohort. Our objective was not to demonstrate the superiority of APRI or PNI over existing established prognostic models. Instead, we aimed to determine their independent predictive value and evaluate their potential complementary role in this clinical setting.

## 2. Materials and Methods

We retrospectively analyzed all consecutive patients with hepatocellular carcinoma (HCC) who underwent hepatic resection at our institution between June 2010 and May 2019. Exclusion criteria were peritoneal dissemination, positive surgical margins, presence of cancer in another organ at the time of surgery, or absence of complete and accurate clinicopathological and follow-up data. Data were prospectively collected and retrospectively reviewed.

None of the patients had coexisting hematologic disorders, ensuring that the platelet counts were representative of normal baseline values. All patients were over 18 years old and had complete clinical and laboratory data available.

Cancer-specific data collected for each patient included clinical, pathological, and surgical variables. The database comprised information on age, sex, pathological prognostic features of the tumor, and preoperative laboratory serum parameters obtained within one month before surgery (including alpha-fetoprotein [AFP], total bilirubin, albumin, aspartate aminotransferase [AST], alanine aminotransferase [ALT], creatinine, hemoglobin, neutrophils, lymphocytes, monocytes, and platelets, Table 1).

Commonly used scoring systems, such as the 8th edition of the American Joint Committee on Cancer (AJCC), Child–Pugh classification, Barcelona Clinic Liver Cancer (BCLC) stage, and Model for End-Stage Liver Disease (MELD) score, were also applied. For major hepatectomies, defined as the removal of ≥3 Couinaud liver segments [18], we assessed the future liver remnant (FLR) volume to evaluate the residual liver volume after resection.

Portal hypertension was defined by indirect signs, including chronic liver disease with endoscopic evidence of esophageal varices, portal hypertensive gastropathy, and/or splenomegaly (defined as a spleen diameter > 12 cm), with or without thrombocytopenia (platelet count < 100,000 cells/mL).

Preoperative imaging findings were obtained from abdominal ultrasound, contrast-enhanced computed tomography (CT), and/or magnetic resonance imaging (MRI), and postoperative pathological data. All patients underwent surgery without prior systemic or local chemotherapy or radiation. Every case was discussed in a weekly multidisciplinary disease team meeting.

Hepatic resection (HR) was considered curative when there was no evidence of distant metastases and complete tumor clearance, both macroscopically and histologically. Removal of hilum lymph nodes was also performed when indicated. Intraoperative liver ultrasound was routinely used.

In this study, the 5-year overall survival (OS) rate was used as the endpoint to stratify the values of various inflammatory indices, including the neutrophil-to-lymphocyte ratio (NLR), platelet-to-lymphocyte ratio (PLR), lymphocyte-to-monocyte ratio (LMR), aspartate aminotransferase-to-neutrophil ratio index (ANRI), fibrinogen-to-albumin ratio (Fib/Alb), systemic immune-inflammation index (SII), prognostic nutrition index (PNI), and the aspartate aminotransferase-to-platelet ratio index (APRI) using receiver operating characteristic (ROC) curve analysis. The optimal threshold values were determined by selecting the cut-off point corresponding to the highest Youden index (specificity + sensitivity − 1). This widely recognized approach ensures the selection of a cut-off point that optimally balances sensitivity and specificity, providing robust discrimination between patient groups and enhancing clinical interpretability. Additionally, liver function was assessed using the albumin–bilirubin (ALBI) score, calculated using the formula: ([log_10_ bilirubin (μmol/L) × 0.66] + [albumin (g/L) × −0.085].

Patients were classified into three ALBI grades based on the following thresholds: Grade 1 (≤−2.60), Grade 2 (>−2.60 to ≤−1.39), and Grade 3 (>−1.39), as defined by previous literature [19].

Furthermore, the PALBI score, which combines platelet count with the ALBI score, was also evaluated as a more precise marker for predicting survival after curative resection [20]. The PALBI score was calculated using the formula: [2.02 × log_10_ bilirubin (μmol/L) − 0.37 × (log_10_ bilirubin)^2^ − 0.04 × albumin (g/L) − 3.48 × log_10_ platelets + 1.01 × (log_10_ platelets)^2^].

Based on this score, patients were classified into three grades: Grade 1 (≤−2.53), Grade 2 (>−2.53 to ≤−2.09), and Grade 3 (>−2.09), with these cut-off points validated in a large population-based study [21].

After discharge, patients were followed up regularly, with visits every three months for the first two years and every six months for the next three years. Follow-up included clinical evaluations and imaging studies, including liver-specific contrast MRI, chest X-ray or CT, and serum AFP levels. Recurrences were confirmed with radiological findings, including contrast enhancement during the arterial phase and early washout in the venous phase.

For patients lost to follow-up, information regarding disease progression or cancer-related death was obtained by contacting their general practitioners or hepatologists.

The study procedures adhered to the institutional ethical standards set by the responsible committee for human experimentation and were conducted in accordance with the 1964 Helsinki Declaration and its later amendments.

### Statistical Analysis

Descriptive statistics were used to summarize the characteristics of the study participants. Continuous variables and their respective ranges were reported as medians, while categorical variables were expressed as frequencies and percentages. For analytical purposes, continuous variables were categorized based on cut-off values identified using receiver operating characteristic (ROC) curve analysis, with overall survival (OS) as the outcome (alive/dead, both overall and within 90 days of surgery) as the state variable. Youden’s index was applied to determine the optimal cut-off value. This index maximized the difference between sensitivity and specificity, as well as the distinction between true positives and false positives.

Differences between variables were evaluated using the Pearson Chi-square test, Fisher’s exact test, or the Mann–Whitney U test, as appropriate. Cox proportional hazard regression models were used to estimate hazard ratios (HRs) and their corresponding 95% confidence intervals (95% CI) in both univariate and multivariate analyses. For each parameter, the most suitable prognostic modality was used as the reference group.

In the multivariate analysis, preoperative and intraoperative parameters that were significant in the univariate analysis were included as potential confounders, using stepwise regression (forward selection), which helps account for multicollinearity and reduce residual confounding. The entry and removal thresholds were set at *p* = 0.05 and *p* = 0.10, respectively. A significance level of *p* < 0.05 was used for all statistical tests. Statistical analyses were performed using SPSS software (version 21, SPSS Inc., Chicago, IL, USA).

## 3. Results

A total of 153 patients with primary hepatocellular carcinoma (HCC) who underwent surgical resection were enrolled in this study. There were 114 men (74.5%) and 39 women (25.5%). The mean age was 69 years (range 33–88 years). The median follow-up duration was 31 months (range 2–109 months). During the follow-up period, 89 patients (58.2%) died. The 1-year, 3-year, and 5-year overall survival (OS) rates were 81.7%, 65.2%, and 40.7%, respectively.

Cirrhosis was present in 127 patients (83%), with the primary cause being hepatitis C virus (HCV) infection (52.9%), followed by hepatitis B, excessive alcohol consumption, and metabolic syndrome. Portal hypertension was reported in 32 patients (20.9%). Of the 153 patients, 147 (96.1%) were classified as Child-Pugh A, and 6 (3.9%) were classified as Child-Pugh B. Among the 153 patients, 146 (95.4%) had a MELD score between 6 and 9, while 7 (4.6%) had a score between 10 and 17. Non-anatomical liver resection (60.1%) was the most common surgical approach, with 86.3% of patients undergoing resection of fewer than three liver segments. Elevated alpha-fetoprotein (AFP) levels (>200 ng/mL) were found in 90 cases (58.8%), and 29 patients (19%) had bilobar tumors. The mean tumor size was 5.03 cm (range 1.4–20 cm), and 57 patients (37.3%) had tumors larger than 5 cm. Macroscopic vascular invasion was found in 23 patients (15%), with portal vein invasion in 10 patients (6.5%) and hepatic vein invasion in 7 patients (4.6%). Portal vein invasion of the main trunk was identified in seven patients (4.6%), with four cases involving the right branch and three involving the left branch. These patients received palliative hepatic resection, whereas the remaining 146 patients (95.4%) underwent curative hepatic resection.

The in-hospital mortality rate was 6.5%, and the mortality rate at 90 days post-surgery was 7%. The clinicopathological data are summarized in Table 2 and Table 3. Additionally, to assess liver function preoperatively, the indocyanine green retention test at 15 min (ICG-R15) was performed on 66 patients, yielding results between 0.3% and 58.3%.

To determine the optimal cut-off points for inflammatory indices in predicting OS after HR for HCC, we analyzed the area under the receiver operating characteristic (ROC) curves. The univariate Cox proportional hazards model identified 18 variables associated with OS. These variables included Child–Pugh B classification (*p* = 0.002), BCLC stage B or C (*p* < 0.001), anatomical resection type (*p* = 0.028), major hepatectomy (*p* < 0.001), tumor size > 5 cm (*p* < 0.001), RBC transfusion (*p* < 0.001), FFP transfusion (*p* = 0.007), postoperative complications (*p* < 0.001), elevated AFP levels (*p* = 0.026), high platelet-to-lymphocyte ratio (PLR) (*p* = 0.035), high aspartate aminotransferase-to-neutrophil ratio index (ANRI) (*p* = 0.043), high fibrinogen-to-albumin ratio (Fib/Alb) (*p* = 0.007), high systemic immune-inflammation index (SII) (*p* = 0.046), low prognostic nutrition index (PNI) (*p* < 0.001), high aspartate aminotransferase-to-platelet ratio index (APRI) (*p* = 0.001), high albumin–bilirubin (ALBI) score (*p* = 0.001), and high PALBI score (*p* = 0.049), all of which predicted poorer OS.

Kaplan–Meier (KM) curve analysis revealed the following 5-year OS rates, stratified by inflammatory markers:

- PLR: 81.1% for ≤145.365, 61.3% for >145.365
- ANRI: 79.3% for ≤13.09, 73.8% for >13.09
- Fib-Alb: 84.7% for ≤71.03, 71.6% for >71.03
- SII: 82.7% for ≤442.455, 65.3% for >442.455
- PNI: 58.1% for ≤41.55, 81.9% for >41.55
- APRI: 80.8% for ≤1.35, 71.4% for >1.35
- ALBI: 85.7% for ≤−2.60, 68.1% for >−2.60 to ≤−1.39, 50.0% for >−1.39
- PALBI: 85.6% for ≤−2.60, 62.4% for >−2.60 to ≤−1.39, 46.9% for >−1.39

Variables identified as statistically significant in the univariate analysis were evaluated in a multivariate analysis (Table 4). The multivariate analysis included Child–Pugh classification, BCLC stage, type of resection, type of hepatectomy, largest tumor size, degree of oncological radicality, RBC and FFP transfusion, postoperative complications, AFP, PLR, ANRI, Fib-Alb, SII, PNI, APRI, and ALBI. The multivariate analysis revealed that the factors significantly impacting OS were BCLC stage, postoperative complications, PNI, and APRI.

**Figure 1:**
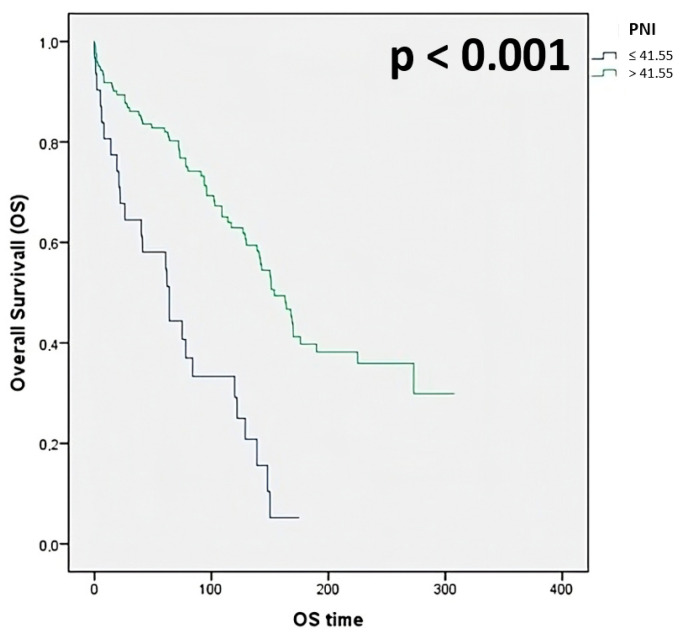
Overall survival stratified for PNI.

Among the inflammatory indices, the PNI and APRI were the only ones that showed a significant prognostic role. Specifically, the postoperative 1-year, 3-year, and 5-year OS rates for the high-PNI group (91.8%, 86.1%, and 82.8%, respectively) were significantly higher than those in the low-PNI group (80.6%, 64.5%, and 58.1%, respectively; *p* < 0.001) (Figure 1).

**Figure 2:**
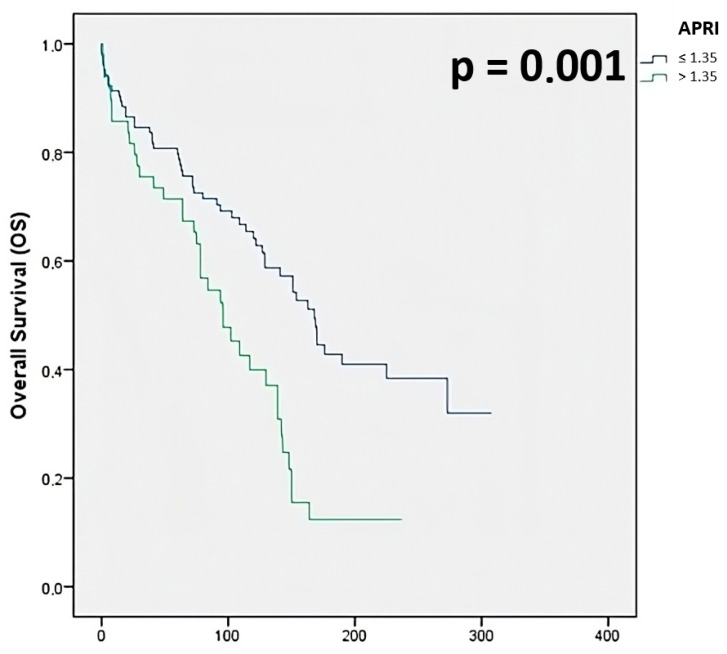
Overall survival stratified for APRI.

Conversely, the 1-year, 3-year, and 5-year OS rates for the high-APRI group (85.7%, 75.5%, and 71.4%, respectively) were significantly lower than those in the low-APRI group (91.3%, 83.7%, and 79.7%, respectively; *p* = 0.001) (Figure 2).

## 4. Discussion

Systemic inflammation is a well-established factor influencing the prognosis of HCC. Over the past fifteen years, several studies have demonstrated that various systemic inflammatory indices are valuable non-invasive tools for predicting survival outcomes in patients with HCC, as well as in those with other malignancies [15, 22–27].

In the present study, we proposed a novel inflammation score system to assess suitable candidates for surgery and to predict overall survival (OS) in patients undergoing hepatic resection (HR). Assessing the preoperative levels of inflammation-related liver disease could therefore help predict varied prognoses following surgery.

A meta-analysis conducted in 2016 showed that NLR could predict survival outcomes in HCC patients undergoing various treatments, including liver transplantation, HR, percutaneous ablation, TACE, and sorafenib [22]. In 2019, a study by Itoh S. et al. reported that low LMR is an independent negative predictor of OS in patients undergoing HR for HCC, suggesting that patients with low preoperative LMR levels tend to have more aggressive tumors [23]. Similarly, a retrospective analysis by Wang Y. et al. confirmed that elevated PLR values predict poor prognosis in HCC patients [24]. In 2012, Pinato DJ et al. first demonstrated that PNI is an independent predictor of OS in HCC patients [10]. Additionally, the APRI score has been highlighted in several studies as a reliable marker for predicting surgical outcomes in HCC patients undergoing HR [25,26].

Our analysis found that high levels of PLR, ANRI, Fib/Alb, SII, APRI, ALBI, and PALBI, as well as low levels of PNI, all predicted poor OS. Kaplan–Meier (KM) curve analyses indicated that patients with high scores in these inflammatory indices had significantly poorer OS compared to those with lower scores. On the other hand, KM analysis for PNI showed that low-PNI scores were associated with significantly poorer OS. These inflammatory indices were reliable predictors of poor prognosis in univariate analysis. However, in multivariate analysis, only PNI, APRI, BCLC stage, and postoperative complications emerged as independent prognostic factors for poor OS, consistent with previous studies [9,10,11,12,13,14,15,25,26,27]. Previous studies, including the work by Yugawa et al. [27], have proposed combining APRI and PNI into a unified prognostic score. In contrast, our study evaluated these indices independently, within a comparative panel of systemic inflammatory markers. Through multivariate analysis, we demonstrated that PNI and APRI emerged as the only independent predictors of overall survival, outperforming other commonly used indices such as NLR, PLR, and SII. These results suggest that even when considered separately, PNI and APRI retain strong prognostic significance and may be readily used in clinical settings without the need for composite scores or complex calculations.

PNI was originally designed to assess the nutritional and immunological status of patients undergoing gastrointestinal surgery [28], and its prognostic significance likely reflects systemic inflammation processes. Although the precise mechanism linking PNI with OS remains unclear, several studies have suggested that lymphopenia is an independent prognostic factor for survival in various cancers. Low-PNI levels may be due to hypoalbuminemia and/or lymphocytopenia, both of which have been linked to impaired immune response and worse cancer outcomes. The depletion of lymphocytes, which play a crucial role in the antitumor immune response, may accelerate tumor progression in HCC [29]. Hypoalbuminemia, a marker of liver dysfunction in chronic liver disease, is also associated with persistent systemic inflammation, which may stem from both the tumor itself and the host’s immune response. Moreover, systemic inflammation has been shown to weaken antitumor immunity, facilitating tumor growth [30].

For APRI, elevated values are associated with poor OS, although the exact mechanism remains unclear. A few hypotheses exist: advanced liver disease may cause mitochondrial damage, leading to the increased release of AST [31,32]. Additionally, the rise in AST levels correlates with liver fibrosis progression, resulting from reduced clearance and mitochondrial injury [33]. The serum AST/ALT ratio, combined with platelet levels, reflects liver inflammatory necrosis, which may promote tumor cell growth. Platelets also play a role in the immune response against tumors, potentially shielding cancer cells from immune surveillance by reducing the cytolytic activity of natural killer cells [34].

The findings of this study support the use of PNI and APRI as practical, non-invasive tools for preoperative prognostic stratification in HCC patients undergoing liver resection. Given their low cost and ease of calculation, these indices can be readily integrated into clinical workflows to identify patients at increased risk of poor survival outcomes. This information may guide more informed surgical decision-making, promote tailored surveillance strategies, and potentially influence the choice of alternative or neoadjuvant treatments in borderline candidates. Importantly, PNI and APRI should complement, rather than replace, established prognostic systems like the BCLC stage, offering an additional dimension of biologically relevant information on host inflammatory and nutritional status [35,36].

Therefore, both PNI and APRI can serve as reliable preoperative tools for evaluating patients with HCC and help stratify prognosis based on varying levels of inflammation, particularly for those undergoing HR.

The cut-off values for inflammatory indices were determined using ROC curve analysis and Youden’s Index. While this is an established and statistically robust method, it is important to consider their clinical interpretability. For instance, a PNI ≤ 41.55 likely reflects a state of poor nutritional and immunological reserve, which is consistent with worse postoperative outcomes. Similarly, an APRI > 1.35 may correspond to advanced liver fibrosis or cirrhosis, conditions known to affect surgical tolerance and long-term prognosis. These thresholds, while data-driven, also appear to align with pathophysiologically meaningful categories.

Nonetheless, the current study has several limitations. First, being a retrospective, single-center study, the predictive power of PNI and APRI for OS requires validation in a larger, multicenter cohort, which limits the generalizability of our findings. Additionally, being a retrospective, single-center study, it may be subject to biases. Moreover, the values of inflammatory indices could be influenced by infections, other underlying inflammatory conditions, or medications, which may affect their prognostic accuracy.

## 5. Conclusions

In conclusion, our study demonstrated that PNI and APRI show promise as clinically useful prognostic biomarkers for overall survival (OS) prediction and for identifying HCC patients who may benefit from hepatic resection (HR). These readily available indices should be considered as complementary tools to established prognostic models, rather than replacements, offering additional insights for patient management and risk stratification. However, further prospective studies are needed to validate their prognostic role, as their predictive accuracy must be confirmed in larger, multicenter cohorts. Future research should aim to include patients who are evaluated by both hepatobiliary surgeons and hepatologists during routine HCC screening. This would help to assess the utility of PNI and APRI across all stages of HCC and provide a more comprehensive validation of their prognostic significance in clinical practice.

## Figures and Tables

**Table 1:** Systemic inflammatory indices evaluated.

NLR	N (absolute neutrophil count)/L (absolute lymphocyte count)
PLR	P (absolute platelet count)/L (lymphocyte count)
LMR	L (absolute lymphocyte count)/M (absolute monocyte count)
PNI	10 × serum albumin (g/dL) + 0.005 × L (lymphocyte count)
SII	P (absolute platelet count) × N (absolute neutrophil count)/L (absolute lymphocyte count)
ANRI	AST (Aspartate Aminotransferase count)/N (absolute neutrophil count)
APRI	[(AST Level IU/L/AST Upper Limit of Normal IU/L)/P (absolute platelet count)] × 100
Fib-Alb	Fibrinogen/Albumin (g/dL) × 100
ALBI	([log_10_ bilirubin (μmol/L) × 0.66] + [albumin (g/L) × −0.085]
PALBI	2.02 × log_10_ bilirubin (μmol/L) − 0.37 × (log_10_ bilirubin)^2^ − 0.04 × albumin (g/L) − 3.48 × log_10_ platelets + 1.01 × (log_10_ platelets)^2^

**Table 2:** Clinicopathologic data, observed 5-year survival rate, and univariate analysis of HCC patients undergoing hepatic resection.

Variable	*n* (%)	5 yrs OS (%)	Exp (B)	95% CI	*p*
Sex Male Female	114 (74.5) 39 (25.5)	78.9 71.5	0.83	0.53–1.31	0.424
Age ≤65 yrs >65 yrs	54 (35.3) 99 (64.7)	77.8 76.7	0.86	0.55–1.34	0.502
ASA score 2 3 + 4	53 (34.6) 100 (65.4)	81.0 74.5	0.36	0.09–1.52	0.163
BMI ^†^ Kg/m^2^ ≤25 >25 < 30 ≥30	78 (51.0) 47 (30.7) 28 (18.3)	74.4 76.6 85.4	0.98	0.74–1.29	0.865
Cirrhosis No Yes	26 (17.0) 127 (83.0)	69.2 78.7	1.10	0.65–1.85	0.720
Portal hypertension No Yes	121 (79.1) 32 (20.9)	75.2 84.4	0.82	0.50–1.36	0.444
MELD score <10 ≥10	146 (95.4) 7 (4.6)	78.6 68.2	0.60	0.32–1.12	0.106
Child-Pugh classification A B	147 (96.1) 6 (3.9)	78.9 33.3	0.26	0.11–0.61	0.002
BCLC stage 0 + A B + C	79 (51.6) 74 (48.4)	88.1 66.2	0.40	0.26–0.61	<0.001
Mini-invasive surgery No Yes	127 (83.0) 26 (17.0)	74.8 88.5	1.45	0.72–2.90	0.297
Type of resection Anatomical Non-anatomical	61 (39.9) 92 (60.1)	65.6 84.8	1.57	1.05–2.42	0.028
Type of hepatectomy Minor (<3 segments) Major (≥3 segments)	132 (86.3) 21 (13.7)	81.8 47.6	0.40	0.24–0.67	<0.001
Largest tumor size ≤5 cm >5 cm	96 (62.7) 57 (37.3)	85.4 63.2	0.47	0.30–0.69	<0.001
Edmondson grading G1–2 G3–4	78 (51.0) 75 (49.0)	85.6 67.1	0.36	0.08–1.64	0.204
Microvascular invasion No Yes	88 (57.5) 65 (42.5)	84.1 67.7	0.79	0.47–1.08	0.106
Capsuled tumor No Yes	99 (64.7) 54 (35.3)	73.7 83.3	1.12	0.72–1.76	0.624
Satellite lesions Negative Positive	139 (90.8) 14 (9.2)	77.0 85.7	0.99	0.50–1.97	0.975
Degree of radicality R0 R1	146 (95.4) 7 (4.6)	78.7 42.9	0.30	0.12–0.77	0.012
Transfusion of RBCs ^‡^ No Yes	116 (75.8) 37 (24.2)	84.3 54.1	0.37	0.24–0.58	<0.001
Transfusion of FFP ^§^ No Yes	137 (89.5) 16 (10.5)	79.4 56.3	0.44	0.24–0.80	0.007
Pringle maneuver No Yes	107 (70.0) 46 (30.0)	77.5 76.1	0.91	0.58–1.42	0.673
Postoperative complication No Yes	79 (51.6) 74 (48.4)	88.6 64.9	0.34	0.22–0.53	<0.001
AFP ^||^ (ng/mL) ≤200 >200	63 (41.2) 90 (58.8)	87.7 57.7	0.53	0.31–0.93	0.026

^†^ BMI: Body Mass Index; ^‡^ RBCs: Red Blood Cells; ^§^ FFP: Fresh Frozen Plasma; ^||^ AFP: alpha-fetoprotein.

**Table 3:** Systemic inflammatory indices of HCC patients undergoing hepatic resection.

Variable	*n* (%)	5 yrs OS (%)	Exp (B)	95% CI	*p*
NLR <2.555 ≥2.555	90 (58.8) 63 (41.2)	80.0 73.0	0.74	0.49–1.12	0.158
PLR ≤145.365 >145.365	122 (79.7) 31 (20.3)	81.1 61.3	0.59	0.37–0.96	0.035
LMR ≤2.96 >2.96	80 (52.3) 73 (47.7)	72.5 82.2	1.10	0.72–1.67	0.688
ANRI ≤13.09 >13.09	92 (60.1) 61 (39.9)	79.3 73.8	0.65	0.43–0.99	0.043
Fib-Alb ≤71.03 >71.03	72 (47.1) 81 (52.9)	84.7 71.6	0.57	0.36–0.85	0.007
SII ≤442.455 >442.455	104 (68.0) 49 (32.0)	82.7 65.3	0.65	0.42–0.99	0.046
PNI ≤41.55 >41.55	31 (20.3) 122 (79.7)	58.1 81.9	3.02	1.89–4.58	<0.001
APRI ≤1.35 >1.35	104 (68.0) 49 (32.0)	80.8 71.4	0.49	0.32–0.75	0.001
ALBI ≤−2.60 >−2.60 ≤ −1.39 >−1.39	77 (50.3) 72 (47.1) 4 (2.6)	85.7 68.1 50.0	0.48	0.31–0.73	0.001
PALBI ≤−2.60 >−2.60 ≤ −1.39 >−1.39	97 (51.6) 48 (48.4) 8 (48.4)	85.6 62.4 46.9	0.65	0.42–0.99	0.049

**Table 4:** Multivariate analysis with Cox Proportional Hazard method.

	HR	95% CI	*p*
BCLC stage	0.38	0.22–0.66	0.001
Postoperative complications	0.48	0.28–0.82	0.007
PNI	2.47	1.33–4.59	0.004
APRI	0.48	0.28–0.81	0.006

## Data Availability

The data supporting this study’s findings are available from the corresponding author upon reasonable request.

## Abbreviations

The following abbreviations are used in this manuscript:

OS	Overall Survival
HCC	Hepatocellular carcinoma
NLR	Neutrophil-to-lymphocyte ratio
PLR	Platelet-to-lymphocyte ratio
LMR	Lymphocyte-to-monocyte ratio
ANRI	Aspartate aminotransferase-to-neutrophil ratio index
Fib-Alb	fibrinogen-to-albumin ratio
SII	Systemic immune-inflammation index
PNI	Prognostic nutritional index
APRI	Aspartate aminotransferase-to-platelet ratio index
LT	Liver transplantation
HR	Hepatic Resection
RFS	Recurrence-free survival
CT	Computed tomography
MRI	Magnetic resonance imaging
AFP	Alpha-fetoprotein
